# Structure of a tryptophanyl-tRNA synthetase containing an iron–sulfur cluster

**DOI:** 10.1107/S1744309110037619

**Published:** 2010-09-23

**Authors:** Gye Won Han, Xiang-Lei Yang, Daniel McMullan, Yeeting E. Chong, S. Sri Krishna, Christopher L. Rife, Dana Weekes, Scott M. Brittain, Polat Abdubek, Eileen Ambing, Tamara Astakhova, Herbert L. Axelrod, Dennis Carlton, Jonathan Caruthers, Hsiu-Ju Chiu, Thomas Clayton, Lian Duan, Julie Feuerhelm, Joanna C. Grant, Slawomir K. Grzechnik, Lukasz Jaroszewski, Kevin K. Jin, Heath E. Klock, Mark W. Knuth, Abhinav Kumar, David Marciano, Mitchell D. Miller, Andrew T. Morse, Edward Nigoghossian, Linda Okach, Jessica Paulsen, Ron Reyes, Henry van den Bedem, Aprilfawn White, Guenter Wolf, Qingping Xu, Keith O. Hodgson, John Wooley, Ashley M. Deacon, Adam Godzik, Scott A. Lesley, Marc-André Elsliger, Paul Schimmel, Ian A. Wilson

**Affiliations:** a Joint Center for Structural Genomics, http://www.jcsg.org, USA; b Department of Molecular Biology, The Scripps Research Institute, La Jolla, CA, USA; c Protein Sciences Department, Genomics Institute of the Novartis Research Foundation, San Diego, CA, USA; d Center for Research in Biological Systems, University of California, San Diego, La Jolla, CA, USA; e Program on Bioinformatics and Systems Biology, Sanford–Burnham Medical Research Institute, La Jolla, CA, USA; f Stanford Synchrotron Radiation Lightsource, SLAC National Accelerator Laboratory, Menlo Park, CA, USA; g Photon Science, SLAC National Accelerator Laboratory, Menlo Park, CA, USA

**Keywords:** TM0492, tryptophanyl-tRNA ligase, tryptophanyl-tRNA synthetase class I, iron–sulfur clusters, structural genomics

## Abstract

A novel aminoacyl-tRNA synthetase that contains an iron–sulfur cluster in the tRNA anticodon-binding region and efficiently charges tRNA with tryptophan has been found in *Thermotoga maritima*. The crystal structure of *Tm*TrpRS (tryptophanyl-tRNA synthetase; TrpRS; EC 6.1.1.2) reveals an iron–sulfur [4Fe–­4S] cluster bound to the tRNA anticodon-binding (TAB) domain and an l-­tryptophan ligand in the active site. None of the other *T. maritima* aminoacyl-tRNA synthetases (AARSs) contain this [4Fe–4S] cluster-binding motif (C-*x*_22_-C-*x*_6_-C-*x*_2_-C). It is speculated that the iron–sulfur cluster contributes to the stability of *Tm*TrpRS and could play a role in the recognition of the anticodon.

## Abstract 2

The crystal structure of tryptophanyl-tRNA synthetase from *T. maritima* unexpectedly revealed an iron–sulfur cluster bound to the tRNA anticodon-binding region.

## 1. Introduction

Aminoacyl-tRNA synthetases (AARSs) covalently append amino acids to their cognate tRNAs. This reaction proceeds in two steps. The first involves the activation of the amino acid by ATP to form aminoacyl-adenylate, which then reacts with its corresponding tRNA to form aminoacyl-tRNA. All organisms possess separate tRNA synthetases for each of the 20 standard amino acids. AARSs are grouped into two classes (classes I and II; Eriani *et al.*, 1990 ▶) based on similarities in their sequences and structures and each group contains ten members. Class I AARSs are mostly monomeric and contain a classic Rossmann nucleotide-fold catalytic domain and two highly conserved sequence motifs ‘HIGH’ and ‘KMSKS’ that are critical for their function. Class II AARSs are structurally distinct from their class I counterparts; instead of the Rossmann fold they contain a central antiparallel β-sheet flanked by α-helices and are mostly dimeric or multimeric. The reactions catalysed by the two classes differ: in class I AARSs the aminoacyl group is coupled to the 2′-­hydroxyl of the tRNA, while in class II AARSs the 3′-hydroxyl is preferred. Tryptophanyl-tRNA synthetase (TrpRS; EC 6.1.1.2) belongs to the class I AARSs and, as its name implies, catalyzes the activation of tryptophan by ATP and the subsequent transfer of the tryptophanyl moiety onto the cognate tRNA.

Here, we report a novel TrpRS from *Thermotoga maritima* that contains a [4Fe–4S] cluster bound to the tRNA anticodon-binding (TAB) domain and an l-tryptophan located in the active site. The *Tm*TrpRS structure was determined using the semi-automated high-throughput pipeline of the Joint Center for Structural Genomics (JCSG; Lesley *et al.*, 2002 ▶) as part of the National Institute of General Medical Sciences’ Protein Structure Initiative (PSI).

## 2. Materials and methods

### 2.1. Protein production and crystallization

*TM0492* (GenBank AAD35577.1; gi:4981003; Swiss-Prot Q9WYW2) was amplified by polymerase chain reaction (PCR) from *T. maritima* MSB8 genomic DNA using *PfuTurbo* (Stratagene) and primers (forward primer, 5′-TTGAGAATACTGAGCGGCATGA­GACC; reverse primer, 5′-gagttaattaattaGAACATCAGGTTCAT­GGCCCTTCTCAC; target sequence in upper case) corresponding to the predicted 5′ and 3′ ends. The PCR product was cloned into plasmid pMH2T7, which encodes a noncleavable expression and purification tag (MGSDKIHHHHHH) at the amino-terminus of the full-length protein. The cloning junctions were confirmed by DNA sequencing. Protein expression was performed in modified Terrific Broth using the *Escherichia coli* methionine-auxotrophic strain DL41. At the end of fermentation, lysozyme was added to the culture to a final concentration of 250 µg ml^−1^ and the cells were harvested. After one freeze–thaw cycle, the cells were sonicated in lysis buffer [50 m*M* Tris pH 7.9, 50 m*M* NaCl, 10 m*M* imidazole, 1 m*M* tris(2-carboxyethyl)phosphine hydrochloride (TCEP)] and the lysate was clarified by centrifugation at 32 500*g* for 30 min. The soluble fraction was applied onto nickel-chelating resin (GE Healthcare) pre-equilibrated with lysis buffer, the resin was washed with wash buffer [50 m*M* Tris pH 7.9, 300 m*M* NaCl, 40 m*M* imidazole, 10%(*v*/*v*) glycerol, 1 m*M* TCEP] and the protein was eluted with elution buffer [20 m*M* Tris pH 7.9, 300 m*M* imidazole, 10%(*v*/*v*) glycerol, 1 m*M* TCEP]. The eluate was diluted tenfold with buffer *Q* [20 m*M* Tris pH 7.9, 5%(*v*/*v*) glycerol, 0.5 m*M* TCEP] containing 50 m*M* NaCl and loaded onto a RESOURCE Q column (GE Healthcare) pre-equilibrated with the same buffer. The protein was eluted with a linear gradient of 50–500 m*M* NaCl in buffer *Q*, buffer-exchanged with crystallization buffer [20 m*M* Tris pH 7.9, 150 m*M* NaCl, 0.5 m*M* TCEP] and concentrated to 18 mg ml^−1^ by centrifugal ultrafiltration (Millipore) for crystallization assays. *Tm*TrpRS was crystallized by mixing 200 nl protein solution with 200 nl crystallization solution and equilibrating against 50 µl reservoir solution in the crystallization plate (Greiner Crystal Quick 96LP) using the nanodroplet vapor-diffusion method (Santarsiero *et al.*, 2002 ▶) with standard JCSG crystallization protocols (Lesley *et al.*, 2002 ▶). The expression and purification tag was not removed from the protein prior to crystallization. The crystallization reagent consisted of 12.5%(*w*/*v*) polyethylene glycol 3000, 0.25 *M* MgCl_2_ and 0.1 *M* cacodylate pH 6.5. A plate-shaped crystal of approximate dimensions 0.15 × 0.10 × 0.02 mm was harvested after 18 d at 277 K for data collection. Glycerol was diluted to 20%(*v*/*v*) using the reservoir solution and then added in a 1:1 ratio to the drop as a cryoprotectant prior to mounting. Initial screening for diffraction was carried out using the Stanford Automated Mounting system (SAM; Cohen *et al.*, 2002 ▶) at the Stanford Synchrotron Radiation Lightsource (SSRL, Menlo Park, California, USA). The diffraction data were indexed in the ortho­rhombic space group *C*222_1_ (Table 1 ▶). The molecular weight and oligomeric state of *Tm*TrpRS in solution were determined using a 1 × 30 cm Superdex 200 column (GE Healthcare) coupled with miniDAWN static light-scattering (SEC/SLS) and Optilab differential refractive-index detectors (Wyatt Technology). The mobile phase consisted of 20 m*M* Tris pH 8.0, 150 m*M* NaCl and 0.02%(*w*/*v*) sodium azide.

### 2.2. Data collection, structure solution and refinement

Native diffraction data were collected on beamline 8.2.2 at the Advanced Light Source (ALS, Berkeley, USA). The data sets were collected at 100 K using an ADSC Quantum 315 CCD detector. Data were integrated and reduced using *XDS* and scaled with the program *XSCALE* (Kabsch, 1993 ▶, 2010*a* ▶,*b* ▶). The structure was determined using the JCSG molecular-replacement pipeline (Schwarzenbacher *et al.*, 2008 ▶) with TrpRS_II from *Deinococcus radiodurans* (*Dr*TrpRS_II; Buddha & Crane, 2005*b* ▶; PDB code 1yi8, chain *B*, sequence identity of 43%) as a search model. The initial solution was found using *MOLREP* (Vagin & Teplyakov, 1997 ▶). Model completion was performed with *Coot* (Emsley & Cowtan, 2004 ▶). TLS refinement was performed with *REFMAC*5 (Winn *et al.*, 2003 ▶; three TLS groups; group 1, residues 0–112; group 2, residues 120–196; group 3, residues 197–328 and the [4Fe–4S] cluster) using a maximum-likelihood target function and individual *B*-factor refinement with appropriate restraints. Residues 113–119 were disordered and were not refined. *CCP*4 programs were used for data conversion and other calculations (Collaborative Computational Project, Number 4, 1994 ▶). Data-processing and refinement statistics are summarized in Table 1 ▶.

### 2.3. Validation and deposition

The quality of the crystal structure was analyzed using the *JCSG Quality Control* server (http://smb.slac.stanford.edu/jcsg/QC). This server processes the coordinates and data through a variety of validation tools including *AutoDepInputTool* (Yang *et al.*, 2004 ▶), *Mol­Probity* (Chen *et al.*, 2010 ▶), *WHAT IF* 5.0 (Vriend, 1990 ▶), *RESOLVE* (Terwilliger, 2003 ▶) and *MOLEMAN*2 (Kleywegt, 2000 ▶), as well as several in-house scripts, and summarizes the results. Protein quaternary-structure analysis was performed with the *PQS* server (Henrick & Thornton, 1998 ▶). The sequence alignment was adapted from an analysis using *ClustalW* (Larkin *et al.*, 2007 ▶) and all other figures were prepared with *PyMOL* (DeLano Scientific). Atomic coordinates and experimental structure factors for *Tm*TrpRS at 2.50 Å resolution have been deposited in the PDB (http://www.pdb.org) and are accessible under code 2g36.

### 2.4. Pyrophosphate-release assays

#### 2.4.1. Aminoacylation assay

Aminoacylation assays were performed with 50 m*M* HEPES pH 7.5, 20 m*M* KCl, 10 m*M* MgCl_2_, 4 m*M* ATP, 1 µ*M* [^3^H]-l-Trp, 19 µ*M* l-Trp, 2 m*M* DTT and 160 µ*M* bulk *E. coli* tRNA. The aminoacylation reactions were initiated by the addition of 100 n*M* enzyme pre-incubated at either 310 or 333 K. Samples were collected at various time points and quenched into a PVDF Multiscreen filter plate containing 100 m*M* EDTA, 300 m*M* sodium acetate pH 3.0 and 0.5 mg ml^−1^ DNA as a carrier. Trichloro­acetic acid was then added to each well at a 10% final concentration to precipitate the tRNA. The plate was then vacuum-dried and washed four times with cold wash solution (5% trichloroacetic acid and 100 m*M* cold l-Trp) to reduce the background radioactivity from free [^3^H]-l-Trp and once with 95% ethanol before scintillation counting.

#### 2.4.2. ATP–PP_i_ exchange assay

PP_i_-exchange reactions were performed in 100 m*M* HEPES pH 7.5, 20 m*M* KCl, 10 m*M* MgCl_2_, 2 m*M* ATP, 2 m*M* sodium PP_i_, [^32^P]-sodium PP_i_, 2 m*M* l-Trp and 5 m*M* β-mercaptoethanol. Reactions were initiated by the addition of 1 µ*M* enzyme pre-incubated at either 310 or 333 K. At each time point, samples were quenched into a PVDF Multiscreen filter plate containing 4% charcoal, 1 *M* HCl, 200 m*M* sodium PP_i_. The charcoal was collected and washed four times with 1 *M* HCl and 200 m*M* sodium PP_i_ prior to scintillation counting.

### 2.5. Comparison with other tRNA synthetases

An iterative *PSI-BLAST* (Altschul *et al.*, 1997 ▶) search was performed for 20 rounds of three iterations each against the NCBI nonredundant (nr) protein-sequence database, using the tryptophanyl-tRNA synthetase sequence from *T. maritima* (gi:4981003) as the initial query. The resulting list of homolog sequences was then queried for the presence of the C-*x*_6_-C-*x*_2_-C pattern from the [4Fe–4S] cluster-binding motif. False-positive hits that contained the Cys pattern at a location other than the TAB region were discarded, resulting in 85 sequences that contained the cluster-binding motif. In addition, a *PSI-BLAST* filtered search using only the C-*x*_6_-C-*x*_2_-C pattern was performed using the sequence of *Tm*TrpRS as a query. An additional 22 unique sequences that were not identified by the previous method were found. Moreover, to ensure that we had exhaustively queried all proteins annotated as tryptophanyl-tRNA synthetases that contained the [4F–4S] cluster-binding motif, a text search was performed in which the nr database was mined for all annotations containing ‘Tryptophan-tRNA synthetase’ and variants thereof. The resulting sequences were then searched for the aforementioned motif of interest. An additional 104 sequences were found using this last approach that were not identified using the previous methods. Although most organisms possess only a single copy of each tRNA synthetase gene, two copies of TrpRS were detected in some bacterial species. The sequences were further analyzed to determine the distribution of the [4Fe–4S] cluster-binding motif. Alignment of the resulting sequences is shown in Supplementary Fig. S1^1^, where only one representative sequence from a clustering at 50% (sequences that are ≥50% identical are clustered as single representative) is shown.

## 3. Results and discussion

### 3.1. Overall structure description

**Figure 1:**
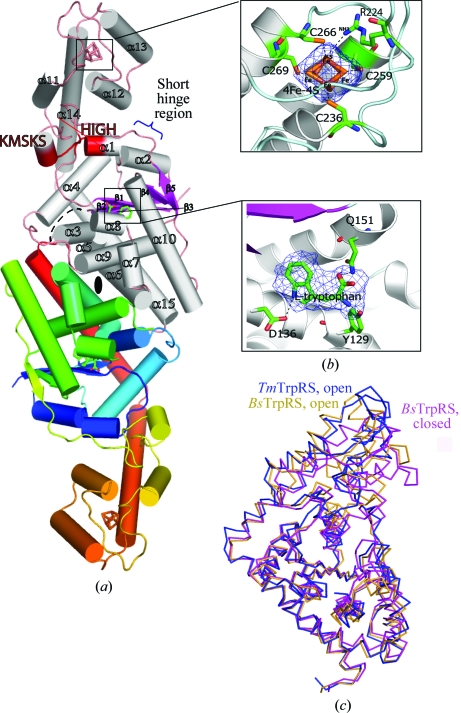
Crystal structure of *Tm*TrpRS. (*a*) Schematic representation of the *Tm*TrpRS crystallographic dimer. The α-helices are represented as cylinders in gray, β-strands as arrows in purple and the loops in pink in one monomer (top) of the dimer. The crystallographically related molecule below is color-coded from blue at the N-­terminus to red at the C-terminus. The bound 4Fe–4S cluster and l-tryptophan are represented as sticks. The β-strands (β1–β5) and α-helices (α1–α15) are labeled (3_10_-helices are not labeled). The ATP-binding motifs (HIGH and KMSKS) are highlighted in red. The short hinge region (residues 182–186) connecting the Rossmann-fold and the TAB domains is indicated. (*b*) Close-up view of a 2*F*_o_ − *F*_c_ OMIT map contoured at 1σ showing the iron–sulfur cluster bound to the cysteine motif [Cys236-*x*_22_-Cys259-*x*_6_-Cys266-*x*_2_-Cys269] in the anticodon-binding region (top box) and the l-tryptophan found in the active site (bottom box). (*c*) Comparison of *Tm*TrpRS to the ‘open’ and ‘closed’ conformations of *Bs*TrpRS.

The 2.5 Å resolution crystal structure of *Tm*TrpRS was determined by molecular replacement (MR) using *Dr*TrpRS_II (PDB code 1yi8; Buddha & Crane, 2005*b* ▶) as a search model. The final model contains one *Tm*TrpRS molecule (residues 0–328), one iron–sulfur cluster [4Fe–4S], one tryptophan and 56 water molecules in the asymmetric unit (Fig. 1 ▶). No interpretable electron density was observed for residues −11 through −1 (from the expression and purification tag) and residues 113–119. The side chains of Lys12, Lys27, Glu74, Thr108, Lys110, Glu111, Glu149, Asp150, Arg182, Lys185, Lys193, Lys209, Glu210, Gu212, Gln213, Lys253, Glu257, Lys271, Lys307 and Lys315 were omitted owing to poor electron density. The Matthews coefficient (*V*_M_; Matthews, 1968 ▶) is 3.49 Å^3^ Da^−1^ and the estimated solvent content is 64.8%. The Ramachandran plot produced by *MolProbity* shows that 97% of the residues are in favored regions, with no outliers.

The *Tm*TrpRS crystal structure, like other bacterial TrpRS structures, contains two domains. The N-terminal catalytic domain adopts a canonical Rossmann nucleotide-binding fold (residues 1–181 and 296–328) with a central, five-stranded, parallel β-sheet (β1–β5; the strand order is 32145) and a tRNA anticodon-binding (TAB) domain that adopts an all-helical fold (residues 187–294) (Fig. 1 ▶). The TAB domain is composed of four α-helices (α11–α14) and two 3_10_ helices (η3 and η4) that are packed as a bundle. A short hinge region (residues 182–186; Fig. 1 ▶ *a*) connects the Rossmann-fold and the TAB domains. The asymmetric unit contains one monomer of *Tm*TrpRS, which forms a crystallo­graphic dimer across the twofold.

As per the SCOP classification (Murzin *et al.*, 1995 ▶), the catalytic domain of TrpRSs belongs to the nucleotidylyl transferase superfamily. While all members of this superfamily retain the core elements of the Rossmann fold, substantial insertions to the catalytic domain, which confer novel functions, have been observed.

**Figure 2:**
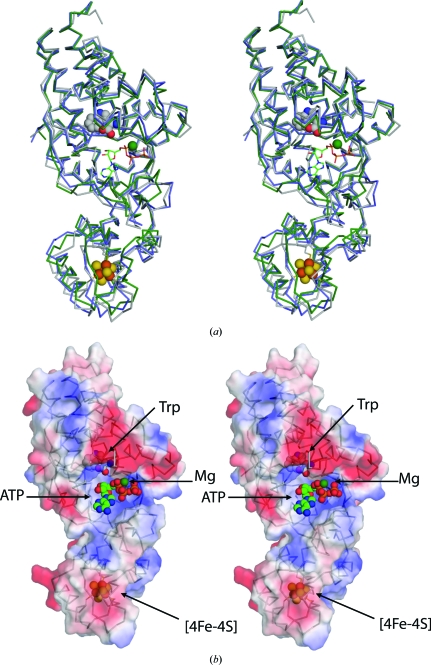
Comparison of *Tm*TrpRS and *Dr*TrpRS_II. (*a*) Structural superposition of *Tm*TrpRS (gray), *Dr*TrpRS_II + ATP and Mg (PDB code 1yid; green) and *Dr*TrpRS_II + Trp (PDB code 1yi8; blue). The bound ATP is shown as a stick model. The [4Fe–4S] cluster (orange and yellow), Mg ion (green sphere) and Trp (gray, red and blue) are shown as spheres. The *Dr*TrpRS_II structures superimpose with *Tm*TrpRS over 319 C^α^ atoms with an r.m.s.d. of ∼1.7 Å (45% sequence identity). (*b*) Electrostatic surface potential of *Tm*TrpRS calculated with the program *APBS* (Baker *et al.*, 2001 ▶): positive potential is shown in blue (+3*kT*e^−1^) and negative in red (−3*kT*e^−1^). l-Tryptophan and the iron–sulfur cluster are shown as spheres. Note: the ATP-binding site is solvent-exposed, but the tryptophan and the iron–sulfur cluster are partially buried.

As expected, a structure-similarity search using *DALI* (Holm & Sander, 1995 ▶) revealed extensive similarities to several class I AARSs. Top *DALI* hits include TrpRS_II from *D. radiodurans* (*Dr*TrpRS_II; Buddha & Crane, 2005*a* ▶; PDB code 1yid; *Z* = 39.7), TrpRS from *Bacillus stearothermophilus* (*Bs*TrpRS; Ilyin *et al.*, 2000 ▶; PDB code 1d2r; *Z* = 22.5) and human tyrosyl-tRNA synthetase (*Hs*TyrRS; Yang *et al.*, 2003 ▶; PDB code 1q11; *Z* = 20.3), among many other class I AARSs. The structural similarity between *Dr*TrpRS-II and *Tm*TrpRS is particularly high and a superimposition of the structures based on secondary-structural elements from both the catalytic Rossmann-fold domain and the C-terminal TAB domain gives an r.m.s.d. of 1.7 Å (Fig. 2 ▶).

Remarkably, the structure of *Tm*TrpRS differs from other TrpRS by the presence of an iron–sulfur cluster [4Fe–4S] in the C-terminal TAB domain. In addition, an l-tryptophan molecule is bound in the active site, which was not expected since tryptophan was not added to any of the reagents used in crystallization or purification.

### 3.2. [4Fe–4S] cluster-binding site

The [4Fe–4S] cluster is chelated by the side chains of Cys236, Cys259, Cys266 and Cys269 from the TAB domain arranged in a C-­*x*_22_-C-*x*_6_-C-*x*_2_-C motif in which four irons are bound to the S atoms of cysteines with distances of 2.28–2.34 Å. The other significant interaction involves the NH1 atom of Arg224 and one of the S atoms (S2) from the cluster, with a distance of 3.31 Å (Fig. 1 ▶ *b*). The presence of the [4Fe–4S] cluster was first identified based on electron density and geometry. The presence of iron in the structure was then con­firmed by X-ray fluorescence scans (Supplementary Fig. S2^1^; for details of how ligands are identified at the JCSG in the course of structure determination, see Kumar *et al.*, 2010 ▶). Mass spectrometry also corroborated the presence of the iron–sulfur cluster. Although *Tm*TrpRS shares extensive sequence similarity in the TAB region with other TrpRSs, the C-*x*_22_-C-*x*_6_-C-*x*_2_-C motif is not found in any other TrpRS present in the PDB. Sequences of *Tm*TrpRS homologs that possess the [4Fe–4S] cluster-binding motif are found in anaerobic organisms from proteobacterial and archaeal groups, but no structures of any of these have yet been reported. *Tm*TrpRS is thus the first reported structure of a TrpRS that contains a [4Fe–4S] cluster.

We extended our search to find other potential iron–sulfur clusters based on the presence of the cysteine-binding motif using *SPASM* (Madsen & Kleywegt, 2002 ▶). For this search, the coordinates of the four cysteines (Cys236, Cys259, Cys266 and Cys269) were used and no substitutions of amino acids were allowed. *SPASM* identified 71 hits representing 22 unique proteins within a 1.5 Å r.m.s.d. of the target motif. The hits included the DNA-repair enzyme endonuclease III (PDB code 2abk; Thayer *et al.*, 1995 ▶), acetyl-CoA synthase (PDB code 1ru3; Svetlitchnyi *et al.*, 2004 ▶) and carbon monoxide dehydrogenase (PDB code 1jqk; Drennan *et al.*, 2001 ▶). The top hit containing the closest structural homolog was the *E. coli* DNA glycosylase MutY (Guan *et al.*, 1998 ▶; PDB code 1mun), which belongs to the DNA-repair enzyme superfamily and excises adenine from mispairs with 8-oxoguanine and guanine. Although the [4Fe–4S] cluster-binding motif (C-*x*_6_-C-*x*_2_-C-*x*_5_-C) of *E. coli* MutY has, in particular, a smaller sequence gap between the first two Cys residues than *Tm*TrpRS (C-­*x*_22_-C-*x*_6_-C-*x*_2_-C), the r.m.s.d. between the two motifs was only 0.88 Å.

**Figure 3:**
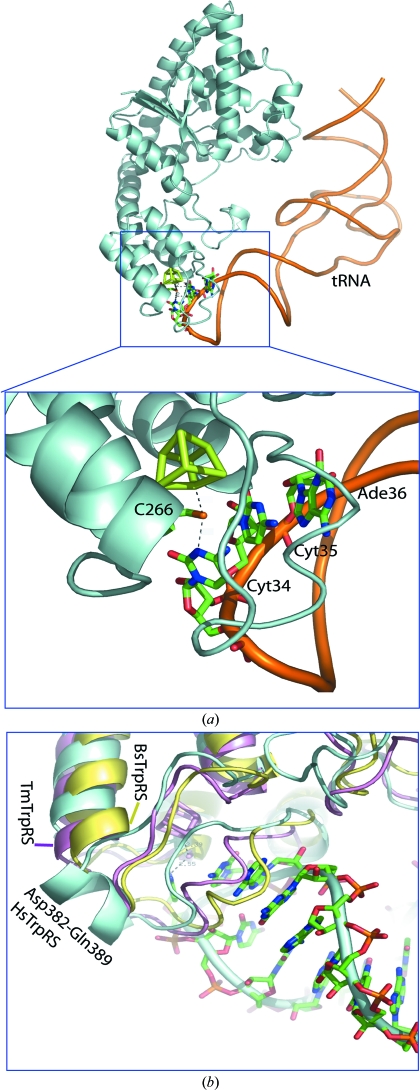
Model of the *Tm*TrpRS–tRNA complex. (*a*) Overall view of the complex. *Tm*TrpRS is shown as a gray-blue ribbon and tRNA as an orange trace. The anticodon bases, Cys266 and the [4Fe–4S] cluster are shown as stick models. Close-up view of the interaction of TrpRS–tRNA with the anticodon (CCA) region. Cyt34 of the tRNA molecule interacts with the iron of the cluster *via* Cys266. The model was based on the structure of the human TrpRS–tRNA complex (PDB code 1r6t). (*b*) Close-up view of the loop conformation of *Tm*TrpRS (pink) near the [4Fe–4S] cluster (pink sticks) compared with the helix (Asp382–Asn389) of the human TrpRS–tRNA complex (gray–blue) and *Bs*TrpRS (yellow).

Comparison of the *Tm*TrpRS structure with that of the human TrpRS–tRNA complex (PDB code 1r6t; Yang *et al.*, 2006 ▶) reveals that, although the sequence identity is very low (19%), the structures are very similar, with an r.m.s.d. of 1.9 Å for 238 superimposed C^α^ atoms. A model of a *Tm*TrRS–tRNA complex based on the complex of human TrpRS with tRNA^Trp^ indicates that Cyt34 of the CCA anti­codon of tRNA^Trp^ can interact with the iron of the cluster *via* Cys266 (Fig. 3*a* ▶).

Interestingly, the *T. maritima* tRNA-modifying enzyme MiaB (*Tm*MiaB), which is involved in the post-transcriptional thiolation and methylation of tRNA, contains an iron–sulfur cluster with a (C-­*x*_3_-C-*x*_2_-C) binding motif (Pierrel *et al.*, 2003 ▶). The iron–sulfur cluster in this case is essential for the modification of the tRNA adenine 37, which helps to stabilize the tRNA anticodon loop. This reaction is catalyzed by the iron that is not coordinated to a cysteine, which is a general theme for iron–sulfur clusters involved in catalysis. In the case of the [4Fe–4S] cluster in *Tm*TrpRS, we believe that it plays a role in the recognition of specifically modified tRNA; however, the biological implications of this are unknown at present. Modifications of the nucleotide in the wobble position 34 are common (Gustilo *et al.*, 2008 ▶); for example, *Saccharomyces cerevisiae* mitochondrial tRNA^Leu^ and tRNA^Trp^ contain a modified U at the wobble position 34 (Martin *et al.*, 1990 ▶). Moreover, 2-thiocytidine is often found in the anticodon loop and all tRNA^Arg^ species from *E. coli* (Jäger *et al.*, 2004 ▶), for example, contain a 2-thiocytidine at position 32.

An interesting difference in the *Tm*TrpRS structure compared with the human TrpRS–tRNA complex is the substitution of helix α17 (Asp382–Gln389) in the human enzyme with a loop in *Tm*TrpRS (Fig. 3 ▶ *b*). As revealed in the crystal structure of the human TrpRS–tRNA complex, α17 is involved in the recognition of the anticodon of the tRNA (Yang *et al.*, 2006 ▶). A similar conformational change has been observed between *Bs*TrpRS and the human enzyme (Fig. 3 ▶ *b*). It was suggested that tRNA binding to *Bs*TrpRS may induce the human enzyme-like conformation in this region (Yang *et al.*, 2006 ▶). Interestingly, the iron cluster is located near this loop. A *PSI-BLAST* search for homologs of *Tm*TrpRS in the NCBI nonredundant (nr) protein-sequence database shows that the C-*x*_(21–­24)_-C-*x*_6_-C-*x*_2_-C motif is mostly found in thermophiles or other extremophiles (Supplementary Fig. 1 ▶ 1). Interestingly, this feature is found in organisms that possess either a single TrpRS gene or multiple genes encoding TrpRS. In those organisms that contain multiple TrpRS genes, only one copy contains the [4Fe–4S] cluster-binding motif (Supplementary Fig. S1^1^).

### 3.3. ATP- and Trp-binding sites

*Tm*TrpRS possesses ATP-binding and Trp-binding sites, which are located close to each other in the Rossmann-fold domain. Typically, this enzyme, which is an obligate dimer, binds ATP and l-tryptophan in one subunit, while the tRNA anticodon region is recognized by the TAB domain from the other subunit. Most class I AARSs are functional as monomers, with the exceptions of TrpRS and TyrRS, which are obligate homodimers. In *Tm*TrpRS, the two relevant subunits pack against each other, burying 2365 Å^2^ of mainly hydrophobic surface. Although a single molecule is present in the asymmetric unit, crystal-packing analysis identified a crystallographic dimer that is likely to represent the biologically relevant dimer. Analytical size-exclusion chromatography in combination with static light scattering indicated that the major species in solution is a dimer.

**Figure 4:**
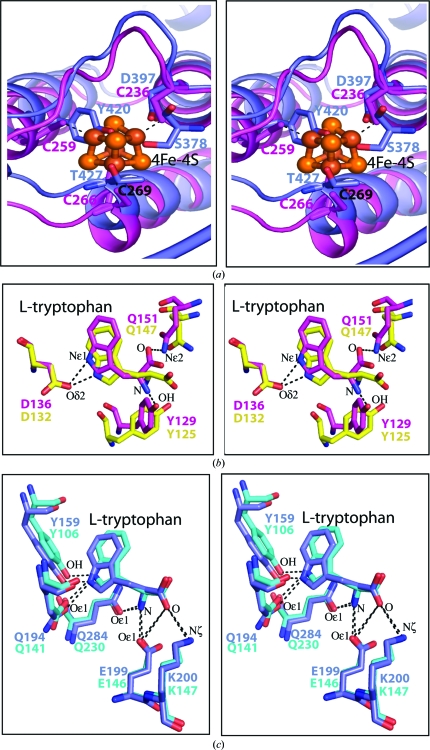
Iron–sulfur cluster and l-tryptophan recognition of *Tm*TrpRS and comparison with other TrpRSs. (*a*) Comparison of the *Tm*TrpRS (magenta) and human TrpRS (blue) TAB domains. The *Tm*TrpRS iron–sulfur cluster is shown in orange ball-and-stick representation. (*b*) Comparison of the l-tryptophan recognition of *Tm*TrpRS (magenta) and *Bs*TrpRS (yellow). (*c*) Comparison of the l-tryptophan recognition of human TrpRS (blue) and *Sc*TrpRS (slate blue).

Although Trp was not present in any of the crystallization reagents, the structure revealed a bound l-tryptophan molecule in the Trp-binding pocket of the active site (Fig. 1 ▶ *a*), suggestive of tight binding of *Tm*TrpRS towards the substrate. The l-tryptophan-binding site in the *Tm*TrpRS structure is similar to that seen in the *Bs*TrpRS and human TrpRS structures (Figs. 4 ▶ *b* and 4 ▶ *c*; see also §3.4), as are the relative orientations of the bound l-tryptophan. However, the l-­tryptophan recognition in *Tm*TrpRS is more akin to that of *Bs*TrpRS than to that of human TrpRS.

The ATP-binding site is located in a positively charged, solvent-exposed cleft located at the junction of the two domains (Fig. 2 ▶). It contains the two signature sequences that are conserved across all members of the class I AARSs: the ^14^HIGH^17^ and ^193^KMSKS^197^ motifs responsible for binding to the adenosine moiety of ATP (Fig. 1 ▶ *a*). The ATP-binding cleft opens and closes *via* a rotation about a hinge between the Rossmann-fold domain and the anticodon-recognition domain (Fig. 1 ▶ *c*). In the *Tm*TrpRS structure, which is similar to the ‘open’ conformation of *Bs*TrpRS, the KMSKS motif is further from the ATP site and is not poised to bind ATP. Accordingly, no ATP molecule was observed in the crystal.

**Figure 5:**
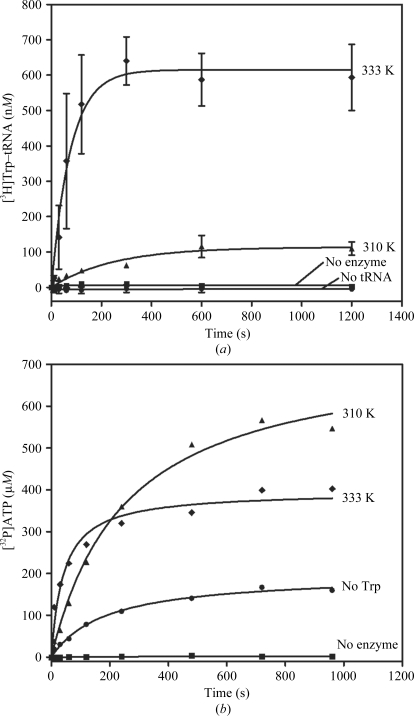
Enzymatic activities of *Tm*TrpRS. (*a*) Aminoacylation activity assayed at 310 and 333 K. Consistent with its thermophilic nature, *Tm*TrpRS has a more robust tRNA-charging activity at 333 K compared with that at 310 K. Control reaction assays lacking enzyme or tRNA at 333 K are also shown. Points are the mean of two assays and error bars represent the standard error of the mean of measurements. (*b*) ATP–PP_i_-exchange activities assayed at 310 and 333 K. This experiment was only performed once. Plots were derived by fitting to an exponential rise to maximum function. Control reaction assays lacking enzyme or Trp at 333 K are also shown. Consistent with the observation of an endogenously bound Trp molecule in the active site of its crystal structure, *Tm*TrpRS had some PP_i_-exchange activity even when no Trp was added to the reaction.

TrpRS activity was confirmed for TM0492. The activity was characterized in tryptophan-dependent ATP–PP_i_ exchange (1) and aminoacylation assays (sum of equations 1 and 2),
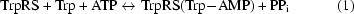

               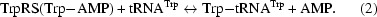
The overall aminoacylation of tRNA^Trp^ can be measured by the incorporation of l-tryptophan into the tRNA to form Trp–tRNA^Trp^ in the presence of ATP. Consistent with its thermophilic nature, *Tm*TrpRS has a more robust tRNA-charging activity at 333 K compared with that at 310 K (Fig. 5 ▶ *a*). The ATP–PP_i_ exchange reaction assesses the reverse of amino-acid activation by measuring the incorporation of [^32^P]-PP_i_ into ATP (1). TM0492 was also active in this assay at both 310 and 333 K (Fig. 5 ▶ *b*). Although the amount of [^32^P]-ATP reached a lower plateau at 333 K than at 310 K, presumably owing to the increase in ATP hydrolysis at higher temperature, the initial PP_i_-exchange rate was higher at 333 K than at 310 K, consistent with the thermophilic nature of TM0492. Furthermore, PP_i_-exchange activity was apparent even when no Trp was added to the reaction (Fig. 4 ▶ *b*). This result is consistent with the observation of an endogenously bound tryptophan molecule in the active site of the crystal structure.

### 3.4. Ligand-binding modes in *Tm*TrpRS and human TrpRS

**Figure 6:**
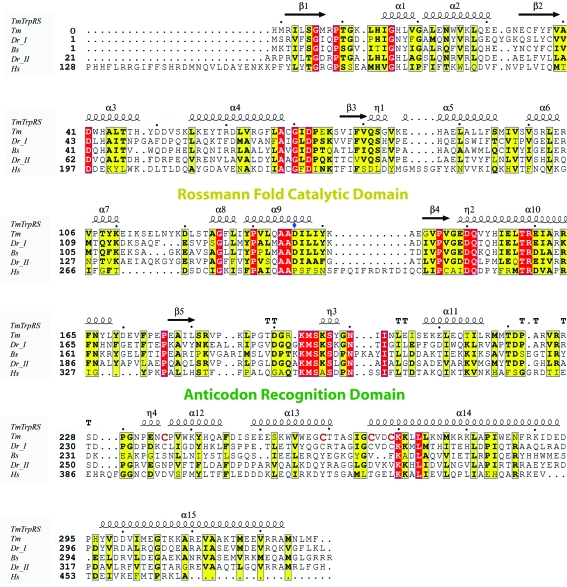
Structure-based sequence alignment of TrpRSs. The four Cys residues in the C-*x*_22_-C-*x*_6_-C-*x*_2_-C motif of *Tm*TrpRS that chelate the [4Fe–4S] cluster are colored by red letters. Strictly conserved residues are highlighted in red boxes and additional conserved residues are in yellow boxes. The secondary-structure elements of *Tm*TrpRS are shown at the top, where α-­helices (α1–α15), β-strands (β1–β5) and 3_10_-helices (η1–η4) are sequentially labeled and β-turns and γ-turns are designated with the Greek letter tau (T). Asp136, which is conserved among prokaryotes, is indicated with a blue diamond. Abbreviations: *Thermotoga maritima* TrpRS, *Tm*TrpRS; *Deinoccoccus radiodurans* TrpRS_I, *Dr_I*; *Bacillus stearothermophilus* TrpRS, *Bs*; *D. radiodurans* TrpRS_II, *Dr_II*; *Homo sapiens* TrpRS, *Hs*. Note: the sequence of *Dr*TrpRS_I is included in the alignment although there is no crystal structure available from the PDB.

Structural comparison of the iron–sulfur cluster region of *Tm*TrpRS with that of human TrpRS shows that the chemical environment in this region is very different. There is nothing in the human enzyme that replaces the iron–sulfur cluster. The four-cysteine motif of *Tm*TrpRS, Cys236, Cys259, Cys266 and Cys269, is not conserved in the human enzyme (Fig. 4 ▶ *a*). The corresponding residues in the human enzyme are Asp397, Tyr420, Thr427 and Leu430, respectively (Fig. 6 ▶). In addition, the side chains of Tyr420 and Thr427 overlap with the iron–sulfur cluster of *Tm*TrpRS (Fig. 4 ▶ *a*).

Structural comparison of the l-tryptophan-binding pockets of *Tm*TrpRS and human TrpRS revealed that the orientations and relative positions of the l-­tryptophans are similar, but the inter­actions between Trp and the binding-pocket residues differ. N^∊1^ of the tryptophan interacts with Asp136 in* Tm*TrpRS (Fig. 4 ▶ *b*). This Asp is conserved among prokaryotes (Fig. 6 ▶). In human TrpRS, N^∊1^ of the tryptophan interacts with Tyr159 and Gln194 (Fig. 4 ▶ *c*). These Tyr and Gln residues are conserved in *S. cerevisiae* TrpRS as shown in the recent structure (PDB code 3kt0; Zhou *et al.*, 2010 ▶). l-Tryptophan recognition in TrpRS is highly conserved among eukaryotes (Zhou *et al.*, 2010 ▶) and the interactions observed in *Tm*TrpRS are conserved among prokaryotes (Figs. 4 ▶ *b* and 4 ▶ *c*).

## 4. Conclusions

We report the first structure of an iron–sulfur cluster-containing tRNA synthetase. Interestingly, this structure also revealed an l-­tryptophan in the active site. The iron–sulfur cluster located in the anticodon-binding region is coordinated by a four-cysteine motif C-­*x*_22_-C-*x*_6_-C-*x*_2_-C. The role of the iron–sulfur cluster is still not clear. The complexity and energetic cost of synthesizing a [4Fe–4S] cluster suggests that it may not be limited to performing a purely structural role.

In a model of the *Tm*TrpRS–tRNA complex based on the structure of the human complex, the [4Fe–4S] cluster is within contact distance of the tRNA anticodon (Fig. 3 ▶ *a*). This implies that the iron–sulfur cluster could be crucial for anticodon recognition by *Tm*TrpRS. One hypothesis is that the [4Fe–4S] cluster could be involved in recognition of a modified tRNA anticodon. However, the modification state of the tRNA^Trp^ anticodon of *Tm*TrpRS–tRNA is not known.

Availability of more sequences and structures of [4Fe–4S] cluster proteins might shed light on the evolutionary relationships of this enzyme. The information presented here, in combination with further biochemical and biophysical studies, should yield valuable insights into the functional role of the enzyme. Additional information about TM0492/*Tm*TrpRS described in this study is available from TOPSAN (Krishna *et al.*, 2010 ▶; Weekes *et al.*, 2010 ▶) at http://www.topsan.org/explore?PDBid=2g36.

## Supplementary Material

PDB reference: tryptophanyl-tRNA synthetase from* T. maritima*, 2g36

Supplementary material file. DOI: 10.1107/S1744309110037619/wd5146sup1.pdf

## Figures and Tables

**Table 1:** Summary of crystal parameters, data-collection and refinement statistics for *Tm*TrpRS (PDB code 2g36) Values in parentheses are for the highest resolution shell.

Space group	*C*222_1_
Unit-cell parameters (Å)	*a* = 122.89, *b* = 152.73, *c* = 53.07
Data collection	λ_1_
Wavelength (Å)	1.0000
Resolution range (Å)	29.64–2.50 (2.59–2.50)
No. of observations	63816
No. of reflections	17636
Completeness (%)	99.3 (97.9)
Mean *I*/σ(*I*)	10.9 (2.3)
*R*_merge_ on *I*†	0.10 (0.64)
Model and refinement statistics	
Resolution range (Å)	29.64–2.50
No. of reflections (total)	17623‡
No. of reflections (test)	894
Completeness (%)	99.3
Data set used in refinement	λ_1_
Cutoff criterion	|*F*| > 0
*R*_cryst_§	0.19
*R*_free_¶	0.26
Stereochemical parameters	
Restraints (r.m.s.d. observed)	
Bond lengths (Å)	0.013
Bond angles (°)	1.60
Average isotropic *B* value, protein (Å^2^)	45.2
Average isotropic *B* value, ligands†† (Å^2^)	58.7
Average isotropic *B* value, water (Å^2^)	41.3
ESU‡‡ based on *R*_free_ value (Å)	0.26
Protein residues/atoms	322/2563
Ligand/atoms	2/23
Solvent molecules	56

† *R*_merge_ = 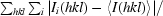 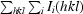.

‡ Typically, the number of unique reflections used in refinement is slightly less than the total number that were integrated and scaled. Reflections are excluded owing to systematic absences, negative intensities and rounding errors in the resolution limits and unit-cell parameters.

§ *R*_cryst_ = 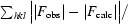 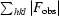, where *F*_calc_ and *F*_obs_ are the calculated and observed structure-factor amplitudes, respectively.

¶ *R*_free_ is the same as *R*_cryst_ but for 5.1% of the total reflections chosen at random and omitted from refinement.

†† L-Trp and iron–sulfur cluster.

‡‡ Estimated overall coordinate error (Collaborative Computational Project, Number 4, 1994 ▶; Cruickshank, 1999 ▶).

## Footnotes

1 Supplementary material has been deposited in the IUCr electronic archive (Reference: WD5146).
