# Supplementary material for: Structure of a tryptophanyl-tRNA synthetase containing an iron–sulfur cluster
Source: Acta Crystallogr Sect F Struct Biol Cryst Commun. 2010 Sep 23;66(Pt 10):1326–34. doi: 10.1107/S1744309110037619 (PMC2954223; doi:10.1107/S1744309110037619)
Supplement: Supplementary file 1 [file f-66-01326-sup1.pdf]

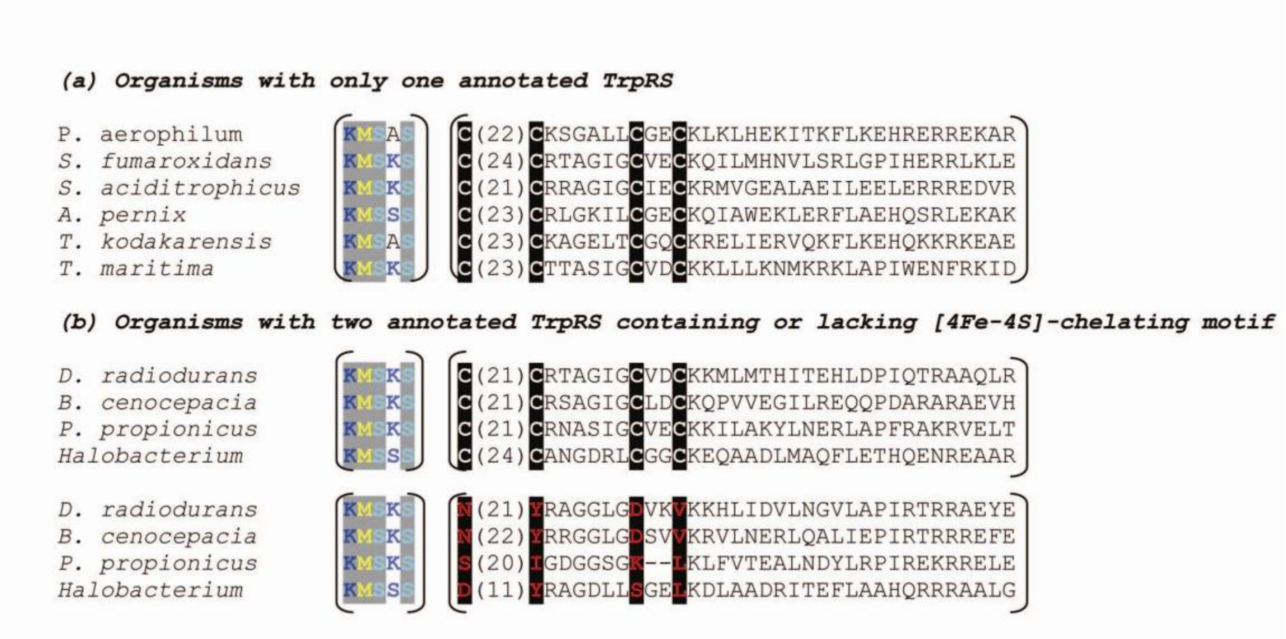

Supplementary Figure S1. Sequence alignment of one or multiple annotated TrpRS in organisms. Only a representative sample from clustering at 50% sequence ID is shown. When the organisms contain two annotated TrpRS, it seems that only one TrpRS gene contains a [4Fe-4S] cluster-binding motif. (Grey columns: conserved “KMS-S” motif; Black columns: four cysteine anti-codon binding region positions.

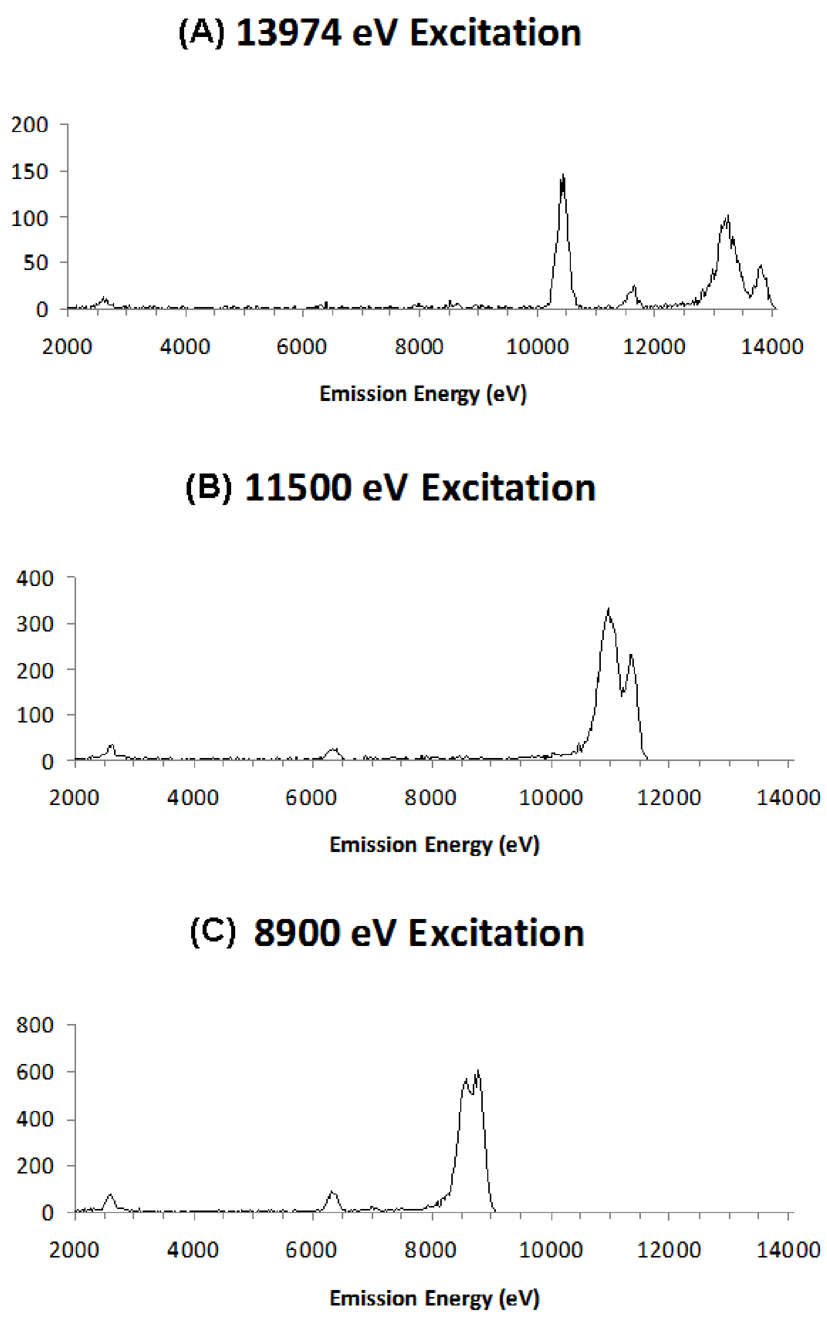

Supplementary Figure S2. X-ray fluorescence excitation scans from the TM0492 crystal used for data collection and structure determination. The spectra show the presence of arsenic, iron and chlorine in the sample. X-ray emission spectra were recorded with a Si-PIN fluorescence detector (Eurisys) and a multi-channel analyzer (Canberra) on SSRL beamline 1-5 using the Blu-Ice data collection control environment. The y-axis shows detector counts. The three scans differ in the energy of the incident X-rays used for excitation: 13974 eV (A), 11500 eV (B) and 8900 eV (C). The elastic and inelastic scattering peaks are the highest energy peaks in each scan and are not fully resolved due to the detector resolution. The 13974 eV scan (A), contains prominent peaks corresponding to the arsenic Kα (10531 eV) and Kβ (11726 eV) emission lines (arising from the 100 mM cacodylate in the crystallization reagent). Iron and chlorine Kα peaks are also visible in this spectra (A) but, due to count rate limitations and the high count rate for the arsenic peak, these peaks are only slightly above the background level. Spectra (B) and (C) were recorded with the incident X-rays below the arsenic absorption edge (11867 eV), which allowed better visualization of the peaks corresponding to Fe (Kα 6400 eV and Kβ 7058 eV) and Cl (Kα 2622 eV and Kβ 2816 eV, which overlap).
